# Mutation load under additive fitness effects

**DOI:** 10.1017/S0016672314000226

**Published:** 2015-02-23

**Authors:** ANDREW C. BERGEN

**Affiliations:** Molecular Genetics and Genomics Program, Washington University in St. Louis, St. Louis, Missouri, 63110, USA

## Abstract

Under the traditional mutation load model based on multiplicative fitness effects, the load in a population is 1−*e^−U^*, where *U* is the genomic deleterious mutation rate. Because this load becomes high under large *U*, synergistic epistasis has been proposed as one possible means of reducing the load. However, experiments on model organisms attempting to detect synergistic epistasis have often focused on a quadratic fitness model, with the resulting general conclusion being that epistasis is neither common nor strong. Here, I present a model of additive fitness effects and show that, unlike multiplicative effects, the equilibrium frequency of an allele under additivity is dependent on the average absolute fitness of the population. The additive model then results in a load of *U*/(*U* +1), which is much lower than 1−*e^−U^* for large *U*. Numerical iterations demonstrate that this analytic derivation holds as a good approximation under biologically relevant values of selection coefficients and *U*. Additionally, regressions onto *Drosophila* mutation accumulation data suggest that the common method of inferring epistasis by detecting large quadratic terms from regressions is not always necessary, as the additive model fits the data well and results in synergistic epistasis. Furthermore, the additive model gives a much larger reduction in load than the quadratic model when predicted from the same data, indicating that it is important to consider this additive model in addition to the quadratic model when inferring epistasis from mutation accumulation data.

## Introduction

The concept of the mutation load first resulted from the insight that in a diploid organism, a locus decreases the average population fitness by an amount double its deleterious mutation rate (Haldane, 1937; Muller, 1950). Under the traditional model of mutation load, which assumes independence among loci, fitness effects combine multiplicatively and result in an average population fitness of *e^−U^*, where *U* is the mutation rate per diploid genome per generation (Crow, 1970). Under high values of *U*, this results in an extremely low average fitness. For example, a recent estimate of *U* in humans is 2·2 (Keightley, 2012), which results in an average fitness of *e^−^*^2.2^, or ~0·11, under this multiplicative model. This average fitness has traditionally been put in terms of offspring viability (Muller, 1950; Nachman & Crowell, 2000). Therefore, the mutation load, *L*, is then 1−*e^−^*^2.2^, or ~0·89, meaning that with selection on offspring viability, 89% of offspring produced die without contributing to the next generation. Consequently, each adult must produce ~9 offspring in order for one to be viable. This reproductive load would actually be ~18 offspring per female, because females must produce both the female and male offspring for each generation. Since populations with low reproductive output, such as humans, do not produce this number of offspring on average, it is apparent that additional factors must be important for explaining how populations deal with such high genomic deleterious mutation rates.

Numerous factors have been brought up as potential explanations for how mutation load may be reduced (reviewed in Reed & Aquadro, 2006; Agrawal & Whitlock, 2012). Notably, selection on fertility, selection on gametes, relative selection, and synergistic epistasis have all been suggested as potential explanations to greatly reduce the load in organisms with low reproductive ability. Under selection on fertility, deleterious mutations act to decrease the total number of offspring an individual can produce, rather than acting to decrease offspring viability. Fertility selection results in a lower reproductive load, but it is dependent on a theoretical, mutation free individual being able to produce a high number of offspring (Lesecque *et al*., 2012). It has been suggested that individuals along the human lineage would have been limited to 11 viable offspring due to physiological constraints, regardless of mutation number, therefore limiting the effectiveness of fertility selection (Lesecque *et al*., 2012). A second explanation is that selection occurs during gametogenesis or on gametes. Selection of this type could purge numerous deleterious mutations with no excess in offspring number, yet it is unknown and questionable how much selection across the genome could occur at these stages (Reed & Aquadro, 2006). Thirdly, in the case of relative selection (which is also referred to as soft selection) an individual's fitness is determined relative to the other individuals in the population (Sved *et al*., 1967; Ewens, 1970; Lesecque *et al*., 2012; Charlesworth, 2013). With relative selection, if the highest fitness is given to the individual in the population with the least deleterious mutations, as opposed to this fitness being given to a theoretical mutation free individual, the load can be greatly reduced. Although some genes may operate under relative selection, others likely reduce fitness regardless of the population average. For example, many mutations may inhibit proper development regardless of other individuals in the population. Overall, the influence of relative selection on the mutation load remains unknown. Although these above factors all have potential in reducing the mutation load to some extent, the focus of this article will be on synergistic epistasis as a means of reducing load, as the additive model presented here falls under this category.

**Fig. 1.**
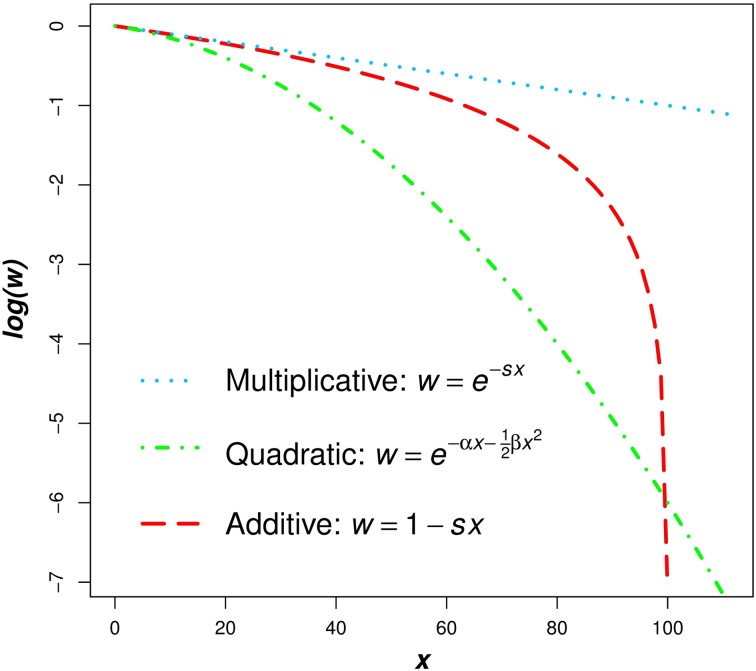
Comparison of the multiplicative, quadratic, and additive fitness functions on a natural-log scale. In these functions, *w* represents fitness and *x* represents mutation number. For the example functions depicted in the graph, *s* and *α* equal 0·01 and *β* equals 0·001. Under the additive model, all individuals where fitness 1*−sx* ⩽ 0 are undefined on a log scale. Because individuals cannot have negative fitness values, individuals where 1*−sx* < 0 are all assigned a fitness of zero in the additive model (see numerical iterations below).

Under synergistic epistasis, as mutation number increases, fitness decreases more than would be predicted under the case of independent, multiplicative effects. Synergistic epistasis results in more deleterious mutations being removed per selective death and consequently the mutation load is reduced (King, 1966; Kondrashov & Crow, 1988). Prominent models of synergistic epistasis include truncation or quasi-truncation selection (Crow & Kimura, 1979; Crow, 1997) as well as a quadratic fitness function (Kimura & Maruyama, 1966; Charlesworth, 1990). Synergistic epistasis can be viewed graphically as a log fitness function that is concave down (e.g. the quadratic fitness function in Fig. 1), whereas the multiplicative fitness model creates a log fitness function that is linear (Fig. 1).

Experiments on model organisms designed to detect epistasis in fitness have detected both synergistic, as well as antagonistic (the opposite of synergistic) epistasis, with the epistasis often being weak and statistically insignificant (reviewed in Kouyos *et al.*, 2007; Halligan & Keightley, 2009; Agrawal & Whitlock, 2012). Specifically, a number of studies have focused on the quadratic fitness model (Kimura & Maruyama, 1966; Charlesworth, 1990) by attempting to detect a significant quadratic term in regressions of log fitness onto mutation number (Mukai, 1969; de Visser *et al*., 1997; Elena & Lenski, 1997; Elena, 1999; Whitlock & Bourguet, 2000). Mukai (1969) detected a significant quadratic term in a regression using *Drosophila* mutation accumulation data, but this result has been questioned due to possible changes in the balancer chromosome, transposable element number, or scoring inconsistencies (Keightley, 1996; Fry *et al.*, 1999). Whitlock and Bourguet (2000) also detected a significant quadratic term, but only with a combination of specific genetic marker mutations. Other regression experiments detected no or very weak and statistically insignificant quadratic terms (de Visser *et al*., 1997; Elena & Lenski, 1997; Elena, 1999). Additionally, most mutation accumulation experiments fit nicely to a linear regression with no significant quadratic term (reviewed in Crow & Simmons, 1977; Halligan & Keightley, 2009). Therefore, the general conclusion from these experiments is that if there is synergistic epistasis, it is weak in general and it is doubtful how much it influences mutation load (Kouyos *et al.*, 2007; Halligan & Keightley, 2009; Agrawal & Whitlock, 2012; Keightley, 2012; Lesecque *et al*., 2012).

However, there is another simple model that is largely absent in current discussions of the mutation load. Namely, mutations decrease fitness additively. In this case, fitness is given by 1*−sx*, where *s* is the selection coefficient of a deleterious mutation and *x* is the number of deleterious mutations an individual possesses. Additivity also gives a log fitness function that is concave down (Fig. 1) and can therefore be considered a model of synergistic epistasis.

With respect to the mutation load, additive effects were first mentioned by Haldane (1937) and Muller (1950), where individual mutation rates per locus, *u*, were simply summed to give a load of 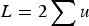, that is, *L* = *U*. However, as stated by Haldane (1937) and Muller (1950), this summation only holds as an approximation when *U* is very small, and no model that maintains accuracy under large *U* was given. Kimura & Maruyama (1966) and Crow (1970) presented a quadratic model of epistasis which, if the quadratic coefficient is set to zero, becomes an additive model. However, Kimura & Maruyama (1966) and Crow (1970) only mention this case briefly and a later analysis continued to use the assumption of *L* = *U* for additive fitness (Crow & Kimura, 1979; see their Discussion specifically). Currently, predictions of *U* in humans are now much larger than one (Eöry *et al.*, 2010; Keightley, 2012), making *L* = *U* an inadequate approximation. Consequently, it remains necessary to provide a derivation of load under additive effects that remains accurate under large *U*.

To accomplish this, I derive the mutation-selection balance equation under additive fitness effects for a single diploid locus and extrapolate this to the entire genome to give the predicted average number of deleterious alleles per individual. These derivations highlight that under additive effects, the average frequency of a deleterious allele is dependent on 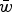, the average absolute fitness in the population, demonstrating why the summation 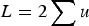 loses accuracy under large *U*. I also perform numerical iterations to determine under what values of *s* my analytic results are approximate.

Additionally, I argue that the common method of inferring epistasis from mutation accumulation data by detecting large quadratic terms from regressions is not necessary, as this additive model is consistent with the data. Specifically, by fitting the multiplicative, quadratic, and additive models to *Drosophila* mutation accumulation data, I demonstrate that though these regressions do not predict large quadratic terms, they fit the additive and multiplicative models equally. Consequently, these data are consistent with the additive model and therefore support a model of synergistic epistasis. I also demonstrate that the additive model can give a larger reduction in load than the quadratic model when predicted from regressions onto the same mutation accumulation data, indicating that it is important to consider this additive model along with the quadratic model when inferring epistasis from mutation accumulation data.

## Theory and models

### Mutation load under multiplicative fitness effects

In this section, I briefly review the standard multiplicative fitness model where independence among sites is assumed. Following Crow and Kimura (1970), consider a locus with two alleles, *A* and *a*, with the following properties

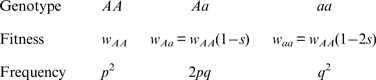

where *s* is the selection coefficient and fitness is in terms of offspring viability. The frequency of *A* is *p* and the frequency of *a* is *q*, where *q* = (1−*p*). The frequency of the wild-type allele *A* in the next generation, *p*′, is equal to
(1)
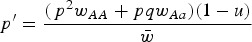

where *u* is the mutation rate from *A* to *a* and 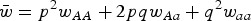 (Crow and Kimura, 1970). At equilibrium, *p* = *p*′ and solving eqn (1) for q gives
(2)
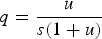

For point mutations, *u* is on the order of 10^−8^ and consequently the term (1+ *u*) in eqn (2) can be ignored to give
(3)
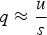

Summing eqn (3) over all *M* loci in a diploid genome where a deleterious mutation can occur gives
(4)
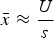

where 
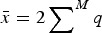
 is the average number of deleterious mutations per individual in the population and 
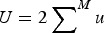
 is the deleterious mutation rate per diploid genome per generation. Note that eqn (4) remains very accurate for varying strengths of *s* as long as *s* is much larger than *u*. However, for the purposes of this discussion it will suffice to assume that all *s* are equal. The average absolute fitness 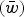 in the population under the multiplicative case is
(5)
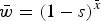

Eqn (5) can be approximated by 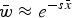 and because of the relationship in eqn (4), can be written as 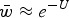. The load, *L*, is equal to 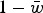 and therefore
(6)
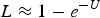

as in Crow (1970). Eqn (6) can be rewritten as 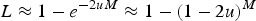, which indicates that the load created by a single locus, *l*, will be *l* = 2*u* (Crow, 1970). Based on this assumption of *l* = 2*u*, previous approximations for load under additive effects were obtained by summing all individual loads across the genome, 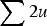, giving the additive approximation of *L* = *U* (Haldane, 1937; Muller, 1950; Crow & Kimura, 1979). However, this approximation is based on the equilibrium frequency given in eqn (3), which assumes multiplicative fitness effects. Therefore, it remains necessary to derive an additive model starting with additive fitness effects.

### Mutation load under additive fitness effects

To derive an additive fitness model, I start with the following values

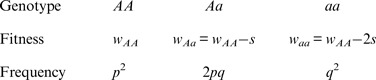


Note that the fitnesses *w*_*Aa*_ and *w*_*aa*_ are found by subtracting an amount from *w*_*AA*_, whereas in the multiplicative case *w*_*Aa*_ and *w*_*aa*_ are found by multiplying *w*_*AA*_ by a percentage. As above, *w*_*AA*_ is the viability of an offspring and *s* is the amount of decrease in viability of an offspring that contains the deleterious mutation. Solving eqn (1) using these additive fitness values gives
(7)
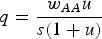

Notice that eqn (7) is the same as eqn (2) except that *w*_*AA*_ remains in the equation. The average absolute fitness in the population, 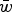, is
(8)
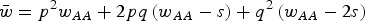

And simplifying eqn (8) gives
(9)
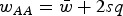

Now, placing the right side of eqn (9) into eqn (7) for *w*_*AA*_ and solving eqn (7) for *q* gives
(10)
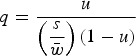


Assuming free recombination between all loci and that the distribution of mutation number is close to Poisson, summing eqn (10) over all *M* loci in a diploid genome where a deleterious mutation can occur, and ignoring the term (1−*u*) because 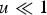 gives
(11)
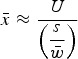

where, as above, 
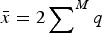
 and 
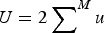
. Notice that eqn (11) is the same as eqn (4) for the multiplicative case except that in eqn (11) *s* is weighted by 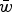. This can be thought of intuitively in that in the multiplicative case, *s* reduces *w*_*AA*_ by a percent equal to (1−*s*). Therefore the ratio 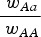 in the multiplicative case is the same for any value of *w*_*AA*_. In contrast, in the additive case *s* reduces *w*_*AA*_ by an amount (not a percent) equal to *s*. In this additive case the ratio 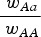 is different for different values of *w*_*AA*_. Other deleterious mutations in the genome lower the average value of *w*_*AA*_ in the population and consequently impact the dynamics at this locus. Again assuming that the distribution of mutation number per individual is close to Poisson, the approximation 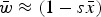 can be used for average fitness. Substituting 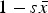 for 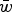 in eqn (11) and rearranging gives
(12)
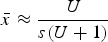


Similarly, since the right sides of eqn (11) and eqn (12) both equal 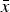, these can be equated and solved for 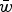 to give
(13)
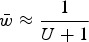


Load in the additive case will be 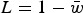, or
(14)
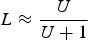


Eqn (14) demonstrates that the load under additivity is greatly reduced under large *U* compared to the assumption of *L* = *U*. These derivations show that it is necessary to take into account the impact that 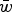 has on the allele frequency, as highlighted in eqn (10), in order to predict the load under additive fitness effects.

### Numerical iterations of the additive model

Numerical iterations were necessary to determine the range of *U* and *s* where eqn (13) above holds as a close approximation for two reason. First, the additive model generates gametic phase disequilibrium (Felsenstein, 1965; Wade *et al*., 2001), which impacts the skewness of the distribution of mutation number. Increased skewness will make the assumption of 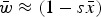 used in the derivations above lose accuracy. Secondly, when deleterious mutations combine additively, all individuals with more than 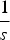 mutations will have a fitness of zero. This truncation point is not accounted for in the analytic derivations above. As either the value of *s* or *U* increases, the probability of an individual being at this truncation point also increases and consequently the average fitness decreases compared to eqn (13). At the extreme, if *s* is equal to 1 (that is, it is lethal), then the average fitness simply becomes *e*^−*U*^, that is, the probability of having zero mutations under Poisson probabilities. Consequently, it is necessary to determine over what range of *U* and *s* the additive model holds.

Details of the iterations are given in the Appendix, but the following gives a brief summary. The iterations assume an infinite population size, with all individuals initially being mutation free. New mutations occur according to Poisson probabilities with an average of *U*. Gametes with *x* mutations are generated based on binomial probabilities assuming free recombination between all loci. Following random mating, offspring survive based upon their viability given additive fitness effects. These generations are iterated until the population reaches a stable fitness value.

**Fig. 2.**
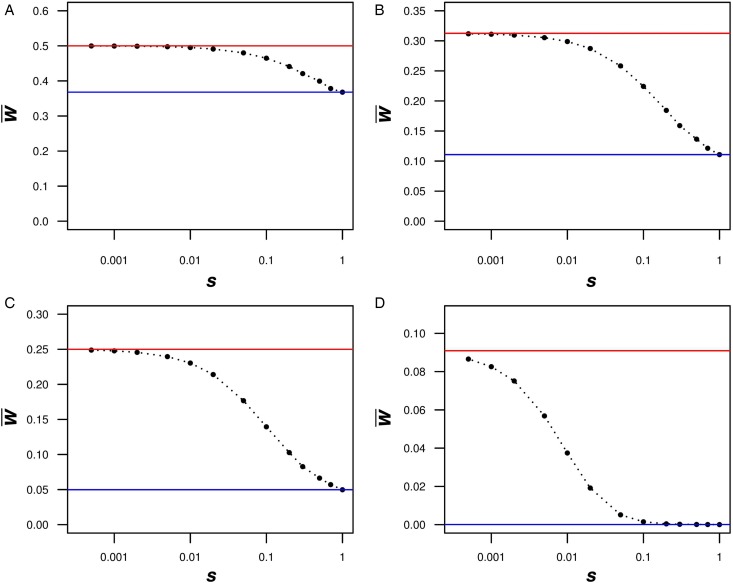
Numerical iterations of absolute fitness for varying degrees of *s* and *U* under additive fitness effects. A. *U* = 1, B. *U* = 2·2, C. *U* = 3, D. *U* = 10. The x-axis represents varying values of the selection coefficient (*s*) on a natural-log scale. The lower straight blue line represents the predicted average fitness 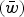 under multiplicative effects (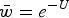) and the upper straight red line represents the predicted average fitness under additive effects 
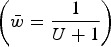
. Each dot represents the equilibrium average fitness from numerical iterations under a given *s*.

The results of the numerical iterations show that eqn (13) holds for small values of *s* (Fig. 2). However, as *s* increases towards 1, it is evident that in all cases absolute fitness approaches *e^−U^*. Moreover, Fig. 2 indicates that as *U* increases, eqn (13) holds for fewer and only smaller values of *s*. Similar patterns are evident for values of 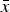 in the iterations compared to eqn (12) above (Fig. S1). These iterations demonstrate that for biologically relevant values of *U* and *s*, the additive analytic predictions fit closely. For example, for *U* equal to 2·2, and an *s* of 0·01, the iteration gives an average fitness of ~0·298, which is very close to 0·3125 predicted from eqn (13).

As stated above, additive effects change the skewness of the distribution of mutation number. In order to visualize this change, the skewness of mutation number per genome (*x*) in the iterations was compared to the skewness predicted under a Poisson distribution (see File S1). These comparisons demonstrate that the additive model distribution of *x* approaches a Poisson distribution as 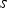 approaches 1 as well as when *s* approaches increasingly small values of *s* (Fig. S2). However, under all values of *U* analyzed, the additive model deviates from a Poisson distribution of *x* for intermediate values of *s* (Fig. S2).

### The additive model fits mutation accumulation data well

The above additive model argues that fitness should decline linearly as mutation number increases. This prediction is consistent with mutation accumulation theory and experiments, which often find good fits for linear regressions, with no log transformation of fitness (Bateman, 1959; Crow & Simmons, 1977; Lynch *et al*., 1999; Halligan & Keightley, 2009 and references therein). In comparison, experiments attempting to identify epistasis by detecting a quadratic term from log-transformed fitness data have also tended to support linear regressions, with no significant quadratic term (de Visser *et al*., 1997; Elena & Lenski, 1997; Elena, 1999; Halligan & Keightley, 2009). Linear regressions likely provide good fits, regardless of whether or not fitness is on a log scale, because these experiments generally do not create individuals with extremely low fitness. As is apparent in Fig. 1, the additive function has a relatively straight line above log(*w*) > −1, that is *w* > 0.37, leaving the additive and multiplicative models indistinguishable if fitness remains high.

As an example of this concept where the additive and multiplicative models can fit data equally, I take three of the earliest *Drosophila* mutation accumulation experiments where no significant quadratic term was detected (Mukai *et al*., 1972). Using the viability data from Mukai *et al*. (1972), regressions of fitness (*w*) onto mutation number (*x*) were performed to predict the multiplicative, additive, and quadratic fitness functions (see File S1 for more details). The functions resulting from these regressions are shown in Table 1 (graphs of these regressions are shown in Fig. S3). As is evident in Table 1, the quadratic regressions give very small quadratic coefficients for all three experiments. *F-*tests demonstrate that there is no statistical significance between the three fits (see File S1 and Table S1). These experiments provide an example where there is equal support for the additive and multiplicative models. Detecting a strong quadratic term is therefore not always necessary for fitting data to a model of synergistic epistasis, as the additive model often provides a good fit.

**Table 1.** Regression equations and numerical iteration values of fitness predicted from the three experiments from Mukai et al. (1972)

Experiment (Mukai *et al.,* 1972)	Fitness model	Function from regression	*R*^2^	Predicted 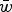 if *U* = 2.2
CH	Multiplicative	ln(*w*) = 0.0022−0.0280*x*	0·995	0·111
	Quadratic	ln(*w*) = 0.0014−0.0271*x−*0.0001*x*^2^	0·995	0·153
	Additive	*w* = 1−0.0255*x*	0·995	0·305
PQ	Multiplicative	ln(*w*) = 0.0022−0.0279*x*	0·993	0·111
	Quadratic	ln(*w*) = 0.0007−0.0245*x−*0.0005*x*^2^	0·995	0·210
	Additive	*w* = 0.9999−0.0254*x*	0·995	0·305
RT	Multiplicative	ln(*w*) = 0.0046−0.0420*x*	0·981	0·111
	Quadratic	ln(*w*) = 0.0037−0.0409*x−*0.0002*x*^2^	0·981	0·149
	Additive	*w* = 1−0.0366*x*	0·980	0·302

The three experiments are labeled CH, PQ and RT in Mukai *et al.* (1972) to represent the three initial fly stocks used to run the three separate mutation accumulation experiments. All values of *R*^2^ and 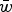 are approximated to three decimal places.

### The additive and quadratic models predict different loads

It is also important to consider an additive model on regression data, and not simply a quadratic model, as a potential epistatic explanation, because these two models will create different reductions in load when predicted from the same data. To demonstrate this difference in load reduction, numerical iterations were run using the predicted multiplicative, quadratic, and additive fitness functions shown in Table 1. These iterations used *U* = 2.2 to be representative of the mutation load for humans if the human fitness model is similar to those predicted by these fly data (see File S1 for details of these iterations). Table 1 demonstrates that the predicted 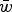 using the additive fitness model are much larger than those predicted using the quadratic model. For example, for experiments CH and RT in Table 1, the predicted fitness using the additive model is twice as large as that predicted from the quadratic model. Consequently, the additive model not only provides a good fit to mutation accumulation data, but also often gives a larger reduction in load compared to the quadratic model.

## Discussion

Additive fitness effects result in a smaller average mutation load compared to multiplicative fitness effects. As stated above, for a predicted *U* of 2·2 in humans, each female would need to produce ~18 offspring for two to survive under the multiplicative model with selection on offspring viability. However, under the additive model presented here, only ~6·4 offspring per female would allow two to survive on average. Data from hunter-gatherer human populations indicate that women who live to the age of 50 have 5·9 live births on average (Eaton *et al*., 1994), making the predicted reproductive load under additive effects very reasonable for populations along the human lineage.

Studies on the distribution of fitness effects predict that the average decrease in fitness due to a deleterious allele, though unknown, is at maximum a few percent, though likely lower (Eyre-Walker & Keightley, 2007). Iterations in Fig. 2 demonstrate that within this range of *s*, around 0·01 or lower, eqn (13) holds as a good approximation for biologically relevant values of *U*.

Mutation accumulation experiments and theory have assumed a linear decline for fitness values that have not been log transformed (Bateman, 1959; Crow & Simmons, 1977; Lynch *et al*., 1999; Halligan & Keightley, 2009), indicating that the additive model provides a good fit to the data in many cases. Interestingly, the quadratic regressions in Mukai (1969) were of viability fitness values that were not log transformed, meaning that even if a quadratic term had not been detected, the regressions would have still supported the additive model, and consequently, synergistic epistasis. Here I demonstrate that the additive, multiplicative, and quadratic models all give regressions for data from Mukai *et al*. (1972) that are not significantly different, indicating that the additive model often has as much support as the multiplicative model. Consequently, detecting a large quadratic term is not always necessary to have support for synergistic epistasis, as the additive model results in epistatic fitness effects. Future experiments focusing on organisms with very low fitness values may help distinguish between these different models, as these low fitness values are where these fitness functions behave most differently.

It should also be noted that there are models of epistasis where declines in fitness do not occur until a certain number of deleterious mutations have accumulated (Crow & Kimura, 1979; Rice, 1998). The existence of such mutations is supported by gene knockout experiments in yeast where gene deletions do not show fitness effects individually, but do show effects in combination (Tong *et al.*, 2004). Mutations which create epistasis in this manner may not necessarily be captured by mutation accumulation experiments. Consequently, there is still the possibility for stronger synergistic epitasis than has been shown in these mutation accumulation studies.

A question that arises is why the additive model has not previously received more consideration as an explanation for mutation load. One factor may be that the original additive summation giving *L* = *U* (Haldane, 1937; Muller, 1950) in a sense gave a solution to load under additive effects. Muller (1950) predicted *U* was much less than 1, making this approximation sufficient. This assumption of *L* = *U* was used in later discussions of fitness under additive effects (Crow & Kimura, 1979; see their Discussion section) and further derivations do not appear to have been pursued. Another factor is that load models came out of population genetics theory, which works most conveniently on a multiplicative scale, as this scale maintains independence among loci. For instance, the quadratic fitness model of Charlesworth (1990) was presented on a log scale so that assumptions about the normality of the mutation distribution could be used. Conversely, load under additive effects is more complex, and requires numerical iterations for exact solutions, making models based on multiplicative effects more mathematically tractable to pursue.

An additional derivation of additive effects was provided to me by Brian Charlesworth (personal communication) which takes into account the departure of the variance of mutation number from Poisson that occurs in one generation of selection (File S2). This derivation gives 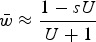, which modifies eqn (13) and helps capture the decrease in average fitness as *s* gets larger (File S2). However, this derivation loses accuracy under large values of *s* as well (Fig. S4), again making the numerical iterations necessary for exact solutions.

Kimura & Murayama (1966) and Crow (1970) presented a quadratic fitness function which is not on a log scale. Consequently, if their quadratic coefficient is zero, the load is *U*/(*U* +1), which is the same as eqn (14) above. However, these authors only mention this case briefly and it was not connected to a model of additive effects, as is done here. Moreover, the average number of mutations per individual in the additive case (eqn (12) above) was not derived in Kimura & Murayama (1966) or Crow (1970). It can however be derived from eqn (1·10) in Kimura & Murayama (1966) by setting their epistatic term (*h*_2_) to zero and solving for the average number of mutations per individual (defined as *λ*). Although these connections to the additive model are present, the discussion put forward here clearly outlines the additive model, how it compares to the multiplicative and quadratic models, and how the average absolute fitness of the population impacts the dynamics of alleles in the case of additivity.

Is there reason to think that fitness effects could be additive? It is important to recognize that although population genetics defines independence among loci as a lack of linkage disequilibrium, quantitative genetics theory defines independence among loci as the absence of interactions among each locus's contribution to a phenotype (Wade *et al*., 2001). Consequently, if each locus contributes to a phenotype independently, then the cumulative phenotype becomes the sum of the contributions from each locus, resulting in additive effects (Fisher, 1918). There is support that in many cases additivity provides an adequate explanation for much of the genetic variance in phenotypes (Lynch & Walsh, 1998; Hill *et al.*, 2008). Furthermore, additive fitness effects are assumed in quantitative genetics studies aiming to detect the additive genetic variance of fitness components in natural populations (Mousseau & Roff, 1987; Kruuk *et al*., 2000; Merilä & Sheldon, 2000; Pettay *et al*., 2005; Teplitsky *et al*., 2009). The assumption of a model of additive fitness effects is not unreasonable. Consider a hypothetical case where multiple loci each independently contribute an amount to an organism's weight. The individual's total weight will naturally be the sum of the contributions from each locus. If the organism's total weight is related linearly to a fitness component, say competition for mating opportunities, then the additive model of fitness would apply. Although such conceptual examples can be given, whether fitness truly behaves on an additive or multiplicative scale remains to be determined.

In summary, additive fitness effects present a simple model that can greatly reduce mutation load without invoking additional factors. Furthermore, additive effects are consistent with analyses of fitness phenotypes based on linear regression. Therefore, although the actual way in which deleterious mutations interact remains unknown, additive fitness provides a model of mutation load that can aid in future theoretical and empirical studies.

## Appendix

### Numerical iterations

Numerical iterations were performed in order to determine exact values of  under specific *U* and *s*. An infinite population size was used where probabilities of mutation, selection, recombination and random mating between genotypes determined the frequency of a genome with *x* deleterious mutations in the following generation. In each generation these occur in the following order: recombination and gamete creation – random mating – selection – mutation – recombination and gamete creation. Then, *x* is counted each generation after mutation in the adult germline genome. The iterations started with an initially mutation free population (that is, the frequency of *x* = 0 is equal to 1).

The following determines the frequency of individuals with *x* mutations in the next generation. Let *i* be the number of new mutations that occur in the adult germline genome, where

is the Poisson probability of an offspring having  new deleterious mutations when the genomic deleterious mutation rate is *U*. An infinite sites model is assumed so that *Pr*(*i*) remains the same regardless of the number of mutations that are already present in the genome. This assumption holds when the number of loci in the genome is much greater than average number of mutations per genome. Also, the Poisson probabilities were truncated at 95 new mutations, because even a under *U* = 10, Pr(*i* = 95) ≈ 10^−58^. Additionally, at a given locus, with , the frequency of a given deleterious allele will be small so that homozygous mutations will be negligible and are consequently ignored in this iteration.

An adult that has *x* mutations will have *i* mutations from new mutations and *x−i* mutations received from its parents. Parent 1 will give a gamete with *j* mutations to this individual (where *j* ⩽ *x−i*). Parent 2 will give a gamete with *x−i−j* mutations. Summing over all gamete combinations, the probability of having a genome with *x* mutations in generation *T* + 1 is

(15)

where

is the probability of Parent 1 giving a gamete with  mutations. Here, *Pr*(*k*)_*T*_ represents the frequency of individuals in the population with *k* mutations in generation *T* and  is the binomial probability of individuals with *k* mutations creating a gamete containing *j* mutations. Binomial probabilities are used to represent free recombination between each mutation. Also, *x*_*max*_ is the maximum number of mutations that an adult germline genome can possess and here is equal to  because offspring with more than  mutations will die and each adult can receive a maximum of 95 new mutations. Similarly,

is the probability of Parent 2 giving a gamete with *x−i−j* mutations. Here, *Pr*(*l*)_*T*_ represents the frequency of individuals in the population with *l* mutations in generation *T* and   is the binomial probability of individuals with *l* mutations creating an gamete with *x*−*i*−*j* mutations. Additionally, in eqn (15), the relative fitness of an individual with *x−i* mutations (*w*_*x*−*i*_) is equal to

where

Similar to above,

and

are the probabilities of parents contributing gametes that contain *n* and *m* mutations, respectively and

The frequencies in generation *T* + 1 for all *x* are generated and these new frequencies are then used to calculate the frequencies of genomes with *x* mutations in generation *T* + 2. These generations are iterated until the average absolute fitness remains constant (to 13 decimals) for 100 generations. The above summations were iterated in Perl.

I would like to thank Justin Fay, members of the Fay Lab, Joe Felsenstein, Brian Charlesworth as well as two anonymous reviewers for helpful comments and suggestions on the manuscript. This work was supported in part by NIGMS Cell and Molecular Biology Training Grant GM:007067 and the NSF (IOS-1339393).

## Supplementary material

For supplementary material accompanying this paper visit https://doi.org/10.1017/S0016672314000226. click here to view supplementary material
