# Supplementary material for: Mutation load under additive fitness effects
Source: Genet Res (Camb). 2015 Feb 23;97:e2. doi: 10.1017/S0016672314000226 (PMC4957254; doi:10.1017/S0016672314000226)
Supplement: Supplementary file 1 [file S0016672314000226sup001.pdf]

Genetics Research
Mutation load under additive fitness effects
Andrew C. Bergen

File S1

Additional Methods

Regressions of mutation accumulation data from Mukai et al. (1972)

Viability measurements of all lines excluding lethal lines were taken from the three experimental lines CH, PQ, and RT as shown in Tables 1, 2, and 3 of Mukai *et al*. (1972), respectively. In the same manner as was done in the plot in Figure 2 of Mukai et al., (1972), viability at each 10 generation period was weighted by the initial viably at generation zero. These experiments measured the mutation accumulation on the *Drosophila* second chromosome and the predicted mutation rate per second chromosome per generation was 0.172. Viability in Mukai et al., (1972) was measured every 10 generations, ending at generation 40. Mutation number for Figure S3 was estimated by multiplying 0.172 by the generation number that viability measurements were taken.

Comparison of the fit of the regressions in Table 1 were done using an *F*-test. This was done in R 3.0.3 using `var.test` on the linear models. The regression on fitness is given by `lm(log(fitness)~mutations)` for the multiplicative case, `lm(fitness~mutations)`for the additive case , and by `lm(log(fitness)~mutations +I(mutations^2))` for the quadratic case. The *P*-values for comparing these 3 models are shown in Table S1.

Measure of skewness created by the numerical iterations

Skewness of deleterious mutation number was measured as a deviation from that predicted under a Poisson distribution (which is the distribution when there is independence among sites). This was accomplished in the numerical iterations by calculating the average number of deleterious mutations per genome ($\bar{x}_{sim}$) for a given U and s. In this case,

$$\bar{x}_{sim} = \sum_{j=0}^{x_{max}} jPr(j)$$

where j is the number of deleterious mutations in an individual and $Pr(j)$ is the frequency of individuals in the iteration at equilibrium with j mutations.

If a distribution is Poisson (as is the case with independence among sites) the skewness should be equal to

$$Predicted\ Skewness = (\bar{x}_{sim})^{-1/2}$$

The actual skew in the distribution at equilibrium was calculated by

$$Actual\ Skewness = \frac{\sum_{j=0}^{x_{max}}(j - \bar{x}_{sim})^3 \Pr(j)}{[\sum_{j=0}^{x_{max}}(j - \bar{x}_{sim})^2 \Pr(j)]^{3/2}}$$

If there is independence among sites, the ratio $Skew\left(\frac{actual}{predicted}\right) = \frac{Actual\ Skewness}{Predicted\ Skewness} = 1$. If the actual skewness of the mutations in the distribution deviates from predicted, the ratio will deviate from 1. Figure S2 shows this ration, $Skew\left(\frac{actual}{predicted}\right)$, for the values of s analyzed in the iterations.

Numerical iterations using regression coefficients from Table 1

Numerical iterations were performed using the fitness functions in Table 1 to predict $\bar{w}$. Iterations were run using the quadratic fitness model

$$w(x) = e^{-\alpha x - \frac{1}{2}\beta x^2} \tag{16}$$

as in Charlesworth (1990), with $\alpha = ah$ and $\beta = 2h^2 b$, where a and b are the linear and quadratic regression coefficients, respectively, and h is the dominance coefficient. Here I use a and b from the quadratic regressions in Table 1, and $h = 0.2$ as in Charlesworth (1990). Similarly, numerical iterations were also run using the additive fitness model

$$w(x) = 1 - sx \tag{17}$$

Here, $s = ah$, where a is the linear regression coefficient from the additive regressions in Table 1. Lastly, numerical iterations were run using the multiplicative fitness model

$$w(x) = e^{-sx} \tag{18}$$

where again $s = ah$ and a is the linear regression coefficient from the multiplicative regressions.

These fitness models in Eqs (16) –(18) were then used to modify w_{x-i} in Eq (15). Specifically, for the quadratic model

$$w_{x-i} = \frac{e^{-\alpha(x-i) - \frac{1}{2}\beta(x-i)^2}}{\bar{w}} \tag{19}$$

where

$$\bar{w} = \sum_{m=0}^{x_{max}} \sum_{n=0}^{x_{max}} \Pr(m) \Pr(n) \left(e^{-\alpha(m+n) - \frac{1}{2}\beta(m+n)^2}\right)$$

and $\Pr(m)$ and $\Pr(n)$ are the same as described above for the numerical iterations. Similarly, for the multiplicative case we have

$$w_{x-i} = \frac{e^{-s(x-i)}}{\bar{w}} \tag{20}$$

where

$$\bar{w} = \sum_{m=0}^{x_{max}} \sum_{n=0}^{x_{max}} \Pr(m) \Pr(n) \left(e^{-s(m+n)}\right)$$

Eq (19) and (20) were used for the value of (w_{x-i}) in Eq (15) and iterations were run with a U of 2.2. For the additive model, s was obtained as described for Eq (17) and used in the additive iterations as described above.

Because Eqs (19) and (20) are fitness functions that never reach a fitness of zero, and therefore the x_{max} in the iterations will approach infinity, a truncation point of 650 mutations per genome was selected where fitness would equal zero. In the iterations the frequency of individuals with 649 mutations was on the order of 10^{-38} or lower, demonstrating that this truncation point does not impact the accuracy of the iteration.

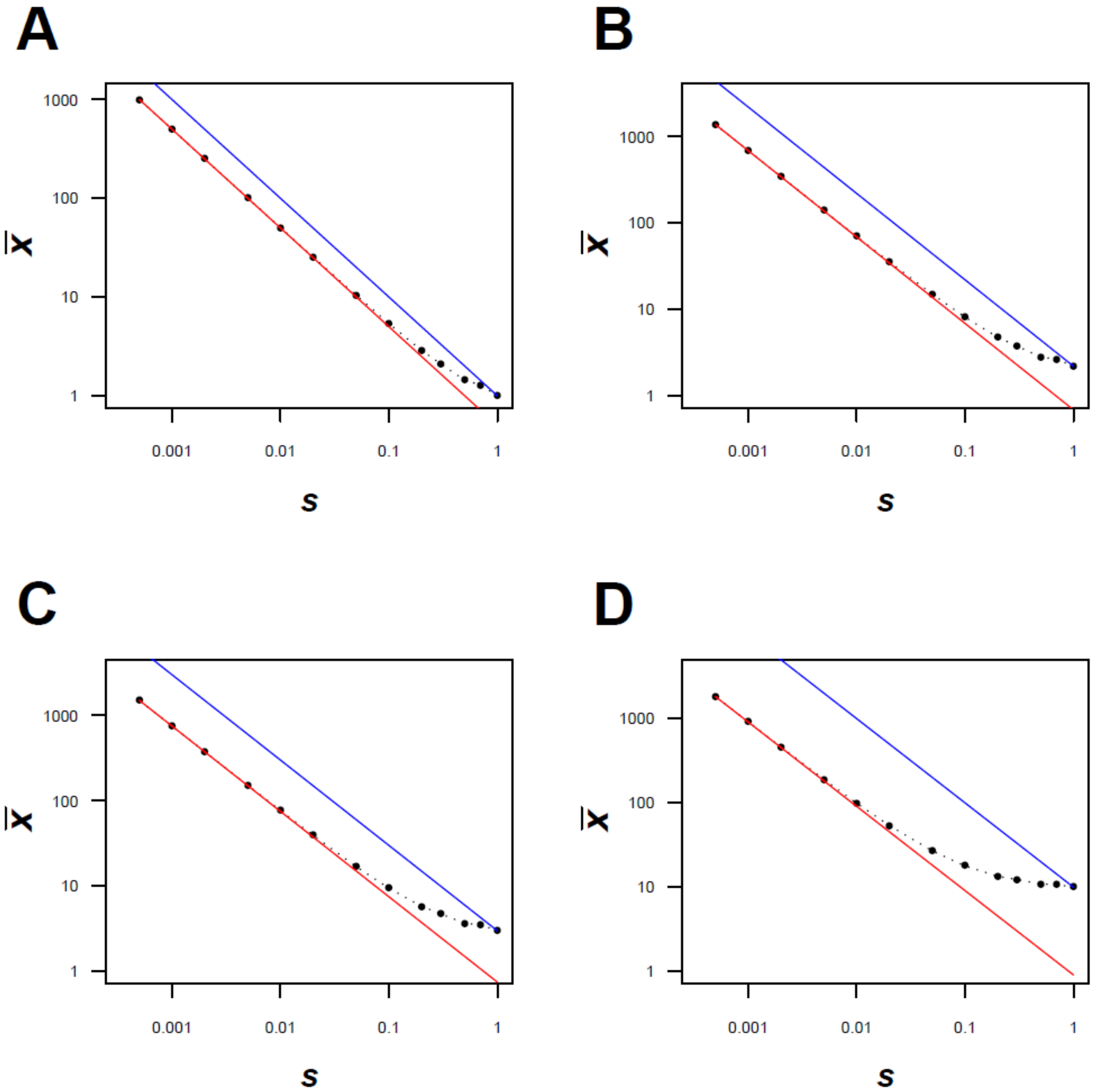

Figure S1. The average number of deleterious mutations per genome, $\bar{x}$, from iterations of additive fitness effects. **A.** U =1 **B.** U =2.2 **C.** U =3 **D.** U =10. The x-axis represents varying values of the selection coefficient (s) displayed on a log scale. Note that $\bar{x}$ is also on a log scale. The higher straight blue line represents the predicted average $\bar{x}$ under multiplicative effects ($\bar{x} \approx \frac{U}{s}$) from Eq (3) and the lower straight red line represents the predicted average fitness under addititve effects $\left(\bar{x} \approx \frac{U}{s(U+1)}\right)$ from Eq (15). Each dot represents the equilibrium average $\bar{x}$ under a given s.

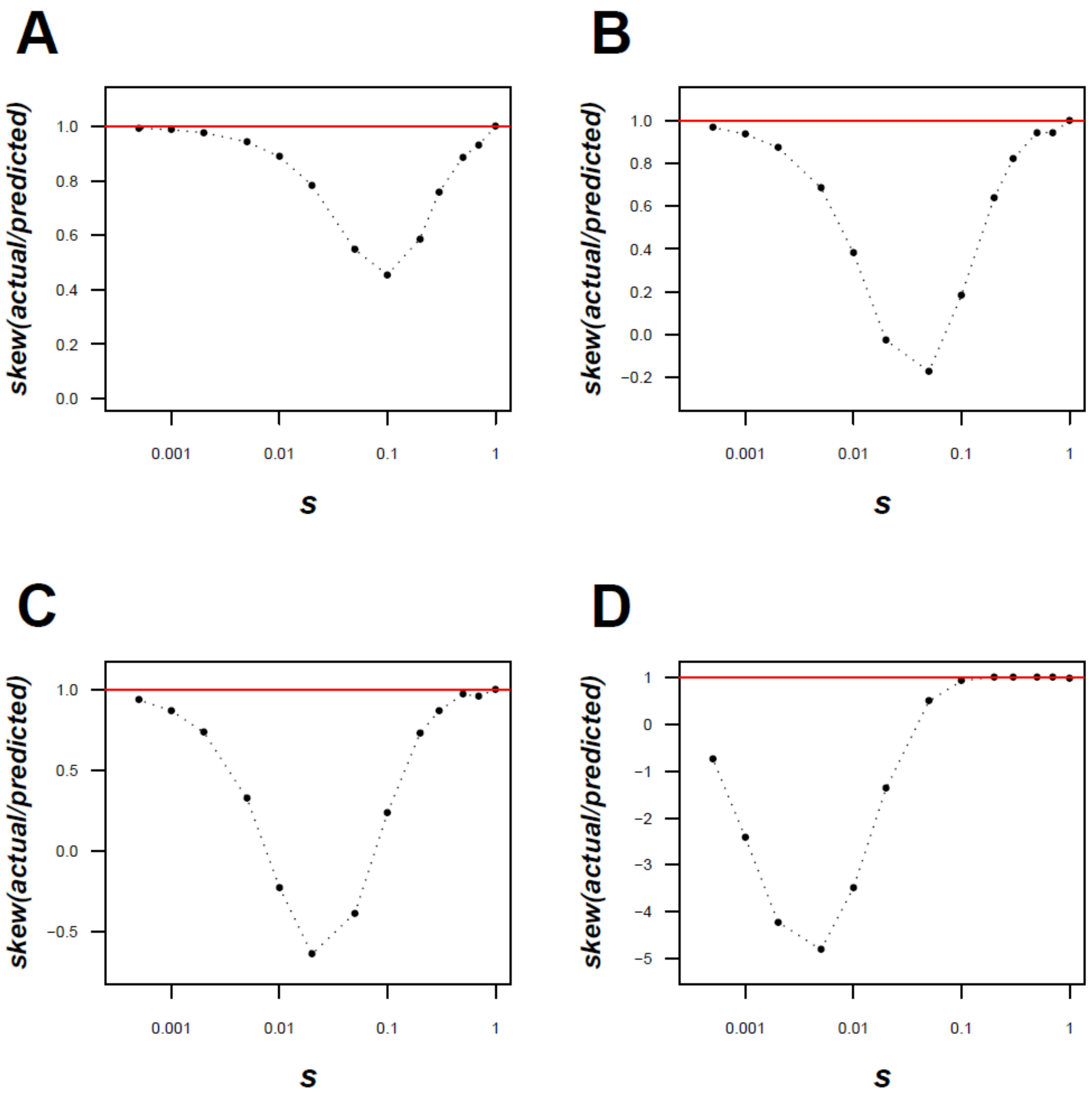

Figure S2. The ratio of the actual skewness of mutation number from the iterations of additive fitness effects, to that predicted from a Poission distribution. **A.** U =1 **B.** U =2.2 **C.** U =3 **D.** U =10. The x-axis represents varying values of the selection coefficient (s) displayed on a log scale. The straight red line represents the ratio $Skew\left(\frac{actual}{predicted}\right) = 1$, and represents the ratio if there is independence among sites.

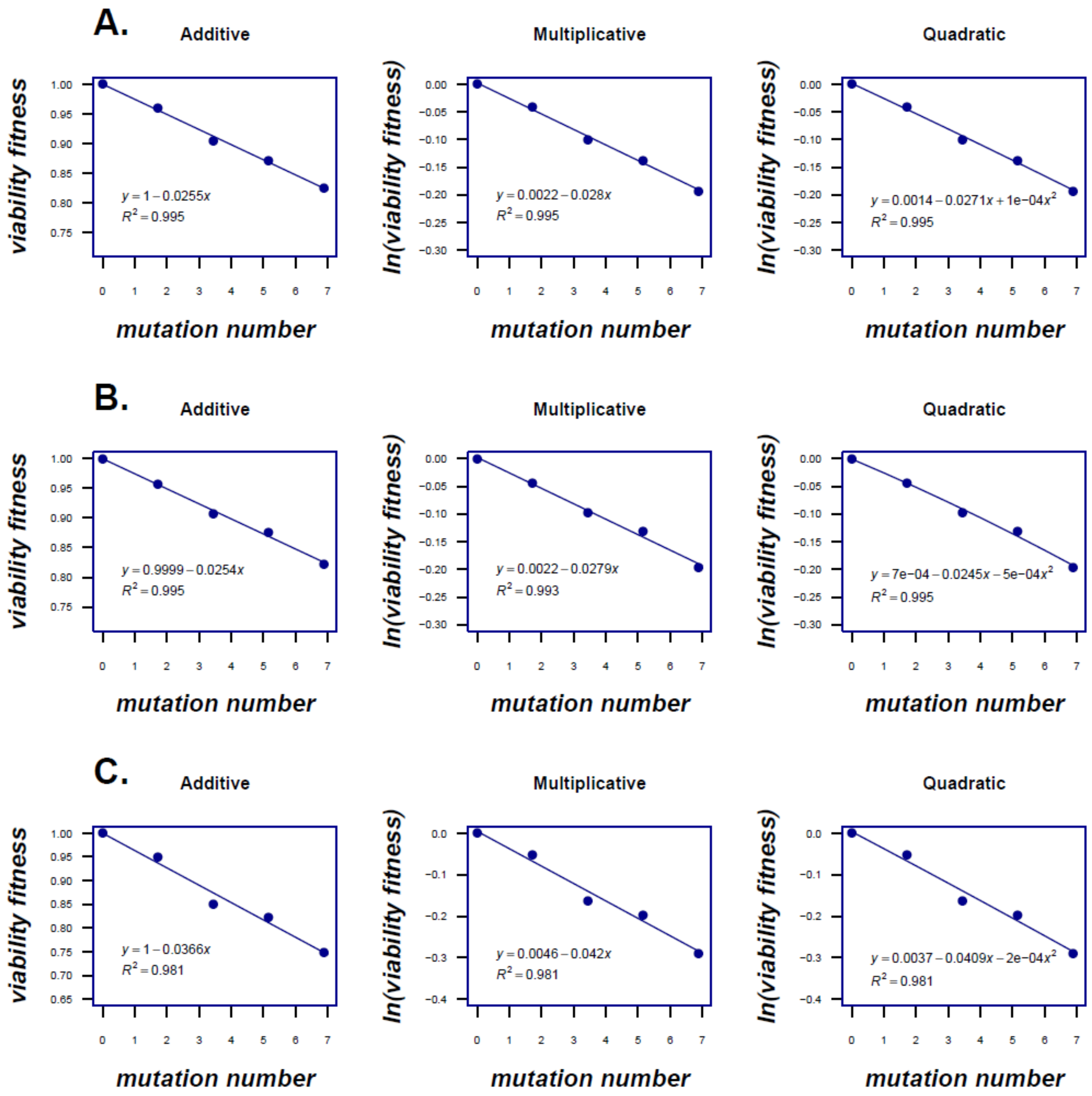

Figure S3. Regressions of *Drosophila* mutation accumulation data from Mukai *et al*. (1972) predicting the additive, multiplicative and quadratic fitness functions. Regressions onto data from **A)** Experiment CH **B)** Experiment PQ and **C)** Experiment RT from Mukai et al. (1972).

Experiment Mukai et al. (1972)	Multiplicative-Quadratic P-value	Multiplicative-Additive P-value	Additive-Quadratic P-value
CH	0.7172	0.9140	0.6464
PQ	0.8245	0.7067	0.5759
RT	0.7087	0.8576	0.5931

Table S1. P-values from comparisons of the regressions for the three models (multiplicative, additive, and quadratic) for each of the three experiments (CH, PQ, and RT) from Mukai et al. (1972). Regressions were compared using `var.test` in R 3.0.3.

File S2

The following alternative method of deriving load equations under additive fitness is based on derivations provided to me by Brian Charlesworth (personal communication). This method takes into account the departure in the variance of mutation number from Poisson that occurs in 1 generation of selection.

Take a trait x, where p(x) is the probability distribution function and follows a standardized Gaussian distribution, with a mean $M[p] = 0$ anda variance $Var[p] = 1$ as in Shnol and Kondrashov (1993). From Eq (2) in Shnol and Kondrashov (1993), the variance of x after selection, $Var[P]$, is

$$Var[P] = \frac{I_2}{I_0} - \left(\frac{I_1}{I_0}\right)^2 \tag{21}$$

where $I_k = \int x^k w(x)p(x)dx$. With additive fitness effects, the fitness function can be defined as $w(x) = a + bx$. The average fitness ($\overline{w}$) is given by I_0 and will therefore be $I_0 = \overline{w} = a$. Additionally, $I_1 = \int xw(x)p(x)dx$ will give $I_1 = b$ and $I_2 = \int x^2 w(x)p(x)dx$ will give $I_2 = a$. Therefore, $Var[P] = 1 - \left(\frac{b}{a}\right)^2$. The change in variance due to selection is equal to $Var[p] - Var[P]$ and since $Var[p] = 1$, this change in variance will be $-\left(\frac{b}{a}\right)^2$. If we define z as the number of mutant genes per genome and V_z as the variance of z, then the change in variance due to selection is $\Delta = -V_z\left(\frac{b}{a}\right)^2$.

The fitness function with respect to z is $w(z) = 1 - sz$. Note that $x = \frac{z-\bar{z}}{\sigma_z}$, where σ_z is the standard deviation of z and $\bar{z}$ is the average. If $w(x) = w(z)$, then $b = -s\sigma_z$ and $a = 1 - s\bar{z}$. This then gives

$$\Delta = -\frac{s^2 V_z^2}{(1 - s\bar{z})^2} \tag{22}$$

Assuming that the distribution of z before selection is Poisson, the variance before selection will be equal to $\bar{z}$. Eqs (9) and (10) in Charlesworth (1990) indicate that Δ represents the departure of the variance from Poisson. That is, $\bar{z} - V_z = \Delta$, so that

$$\bar{z} - V_z = \frac{s^2 V_z^2}{(1 - s\bar{z})^2} \tag{23}$$

Again assuming a normal distribution, we know that the average change in fitness due to selection is

$$\Delta\overline{w} = \frac{V_w}{\overline{w}} \tag{24}$$

in accordance with Fisher's fundamental theorem of natural selection. Because $V_w = s^2 V_z$ and as above $\bar{w} = (1 - s\bar{z})$ so that $\Delta\bar{w} = s(\Delta\bar{z})$. Eq (24) can then can be written as

$$\Delta\bar{z} = \frac{sV_z}{1 - s\bar{z}} \tag{25}$$

which represents the decrease in average number of deleterious mutations per individual due to selection. At equilibrium, the decrease in deleterious mutations due to selection will be equal to the increase due to new mutations, so that

$$\frac{sV_z}{1 - s\bar{z}} = U \tag{26}$$

Substituting (26) into (23) gives $\bar{z} - V_z = U^2$. Therefore, replacing V_z in (26) with $\bar{z} - U^2$ and solving (26) for $\bar{z}$ gives

$$\bar{z} = \frac{U(1 + sU)}{s(1 + U)} \tag{27}$$

and therefore $\bar{w}$ is

$$\bar{w} = 1 - s\left(\frac{U(1 + sU)}{s(1 + U)}\right) = \frac{1 - sU}{1 + U} \tag{28}$$

Eqs (27) and (28) are similar to (12) and (13) in the main text except with the addition of $+sU$ and $-sU$, respectively. Eq (28) helps capture the decrease in fitness that occurs as the value of s increases. This derivation assumes that the distribution of mutation number is Guassian, even after selection. However, as the numerical iterations indicate (Figure S2), additive effects create skewness in the distribution. Also, similar to the derivations for (12) and (13), these derivations also ignore the effect of the truncation point where $1 - s \leq 0$ gives a fitness of zero. As can be seen in Figure S4, though fitness decreases with increasing s using (28), like Eq (13), it also quickly loses accuracy under larger s.

References

Shnol, E.E., and A.S. Kondrashov. 1993. The effect of selection on the phenotypic variance. Genetics 134: 995-996.

Charlesworth, B. 1990. Mutation-selection balance and the evolutionary advantage of sex and recombination. Genet. Res. 55: 199-221.

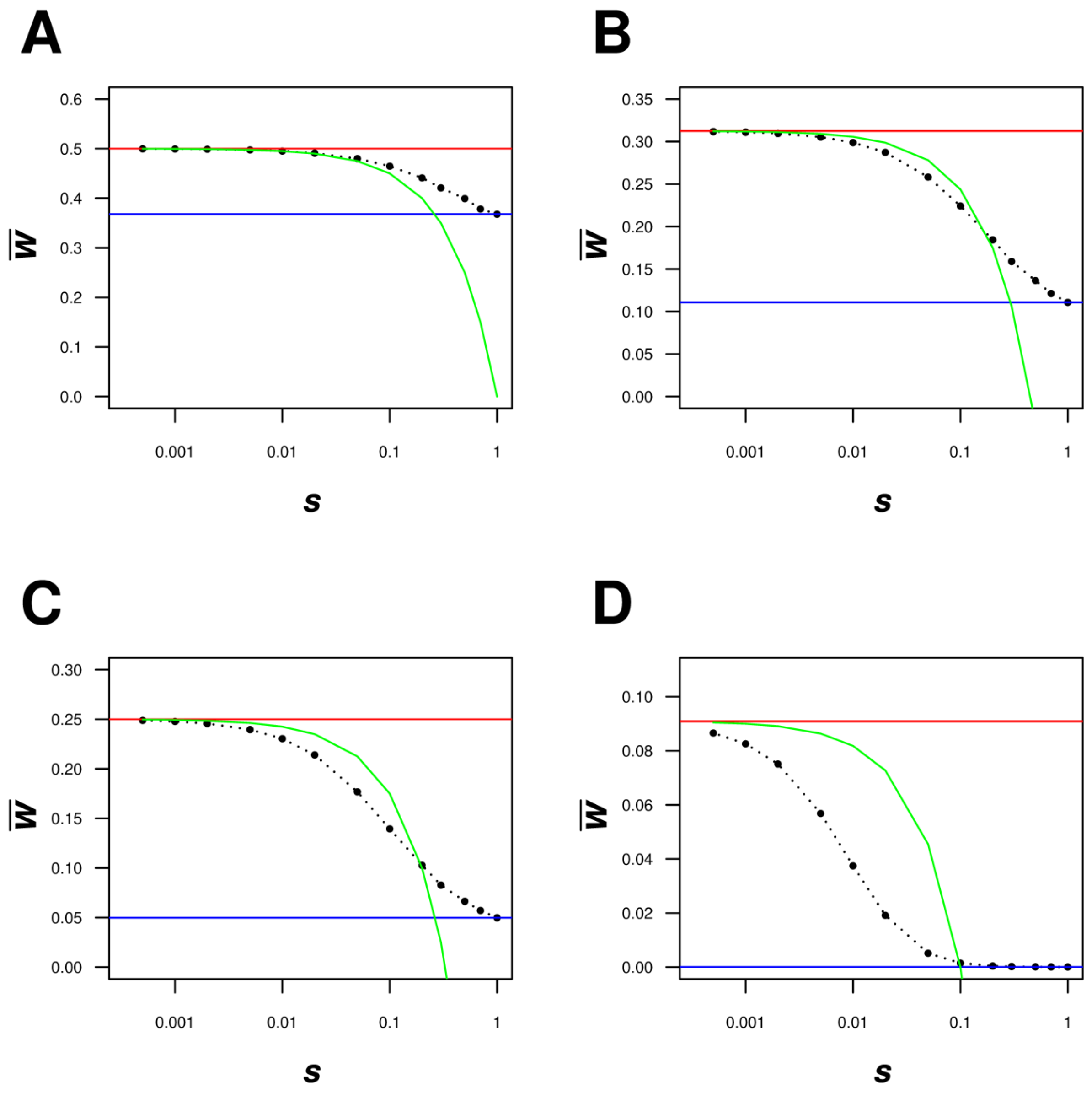

Figure S4. Comparison of Eq (28), where $\bar{w} = \frac{1-sU}{U+1}$, to numerical iterations of absolute fitness for varying degrees of s and U under additive fitness effects. **A.** U =1 **B.** U =2.2 **C.** U =3 **D.** U =10. Similar to Figure 2, the x-axis represents varying values of the selection coefficient (s) on a natural-log scale. The lower straight blue line represents the predicted average fitness ($\bar{w}$) under multiplicative effects ($\bar{w} = e^{-U}$) and the upper straight red line represents the predicted average fitness under additive effects $\left(\bar{w} = \frac{1}{U+1}\right)$ as in Eq (13). Each dot represents the equilibrium average fitness from numerical iterations under a given s. The function in green represents $\bar{w} = \frac{1-sU}{U+1}$ as in Eq (28) in the derivation in File S2.
